# Evaluation of Biological Activities of Twenty Flavones and In Silico Docking Study

**DOI:** 10.3390/molecules28062419

**Published:** 2023-03-07

**Authors:** Meriam Belaiba, Sarah Aldulaijan, Sabri Messaoudi, Manef Abedrabba, Adnene Dhouib, Jalloul Bouajila

**Affiliations:** 1 Laboratoire de Génie Chimique, Université de Toulouse, CNRS, INP, UPS, F-31062 Toulouse, France; 2 Laboratoire des Matériaux Molécules et Applications, Université Tunis Carthage, IPEST, La Marsa 2070, Tunisia; 3 Chemistry Department, College of Science, Imam Abdulrahman Bin Faisal University, P.O. Box 1982, Dammam 31441, Saudi Arabia; 4 Department of Chemistry, College of Science, Qassim University, Buraidah 51452, Saudi Arabia

**Keywords:** flavones, anti-α-amylase, anti-inflammatory (**1**5-lipoxygenase), XOD, molecular docking, structure-activity in silico, cytotoxicity

## Abstract

This work aimed to evaluate the biological activities of 20 flavones (M1 to M20) and discuss their structure–activity relationships. In vitro assays were established to assess their numerous biological activities (anti-α-amylase, anti-acetylcholinesterase, anti-xanthine oxidase, anti-superoxide dismutase, and anticancer cell lines (HCT-116, MCF7, OVCAR-3, IGROV-1, and SKOV-3 cells lines)). An in silico docking study was also established in order to find the relationship between the chemical structure and the biological activities. In vitro tests revealed that M5 and M13 were the most active in terms of anti-α-amylase activity (IC_50_ = 1.2 and 1.4 µM, respectively). M17 was an inhibitor of xanthine oxidase (XOD) and performed better than the reference (allopurinol), at IC_50_ = 0.9 µM. M7 presented interesting anti-inflammatory (IC_50_ = 38.5 µM), anti-supriode dismutase (anti-SOD) (IC_50_ = 31.5 µM), and anti-acetylcholinesterase (IC_50_ = 10.2 µM) activities. Those abilities were in concordance with its high scavenging activity in antioxidant ABTS and DPPH assays, at IC_50_ = 6.3 and 5.2 µM, respectively. Selectivity was detected regarding cytotoxic activity for those flavones. M1 (IC_50_ = 35.9 µM) was a specific inhibitor to the MCF7 cancer cell lines. M3 (IC_50_ = 44.7 µM) and M15 (IC_50_ = 45.6 µM) were particularly potent for the OVCAR-3 cell line. M14 (IC_50_ = 4.6 µM) contributed more clearly to inhibiting the colon cancer cell line (HCT116). M7 (IC_50_ = 15.6 µM) was especially active against the ovarian SKOV human cancer cell line. The results of the biological activities were supported by means of in silico molecular docking calculations. This investigation analyzed the contribution of the structure–activity of natural flavones in terms of their biological properties, which is important for their future application against diseases.

## 1. Introduction

Flavonoids contribute to numerous biological activities through their antioxidant, antimicrobial, anti-allergenic, anti-viral, and anti-inflammatory properties [1]. Their applicability to cancer and Alzheimer’s disease treatments is also being considered [2]. Numerous anti-hyperglycemic drugs are used in monotherapy or in combination therapy, which commonly involves α-amylase inhibitors (AAIs) [3]. Using those AAIs is, however, highly expensive and also has side effects on the organism. Indeed, the inhibitory action of acarbose against pancreatic α-amylase-bound starch digestion causes unpleasant gastrointestinal consequences, such as the increased production of gases, flatulence, abdominal pain, and diarrhea. These discomforts can cause the suspension of AAI therapy by patients [3,4]. Therefore, several studies are now focusing on natural substances from plants that are used in traditional medicine for anti-hyperglycemic treatment [5,6].

Xanthine oxidase (XO) is an important enzyme catalyzing the hydroxylation of hypoxanthine to xanthine and xanthine to uric acid, which is excreted by the kidneys. Excessive production and/or inadequate excretion of uric acid results in hyperuricemia [7]. The inhibition of XOD activity reduces both vascular oxidative stress and the circulating levels of uric acid. Some natural compounds that are present in plants as phenolics have been reported as XOD inhibitors [7,8]). Alzheimer’s disease (AD) is the most common single cause of dementia in our aging society. A total understanding of the mechanism of this disease remains elusive. In the literature, it has been suggested that the β-amyloid protein, abnormal tau protein, or probably both are key factors in the development of AD. At present, the only group of drugs currently authorized for AD treatment is acetylcholinesterase inhibitors. This enzyme controls acetylcholine levels in the neuronal synapses. It has also been also demonstrated that acetylcholinesterase (AChE) is involved in the development of amyloid plaques. Progress in AChE inhibitor drugs followed the finding that cholinergic pathways in the cerebral cortex and basal forebrain are conceded in AD [9].

Owing to toxicity and the severe damage produced in cancer therapy when using non-natural agents, many studies are now focusing on natural compounds with anticancer potential [10,11]. Several important anticancer bio-substances (e.g., paclitaxel, docetaxel; vinblastine, vincristine, topotecan, irinotecan, etoposide, etc.) are being used to treat cancer [12,13,14]. With their anti-cancer specificity, flavonoids have great potential in terms of treatment, and they do not exhibit any considerable post-toxicity consequences [15]. 

In this study, the biological activities of 20 flavones are examined: (i) their contribution to anti-diabetic activity, (ii) the identification of acetylcholinesterase flavone inhibitors that are correlated to AD, (iii) their involvement in anti-xanthine oxidase (XOD) activity, (iv) their SOD inhibition ability, (v) the results of 3-(4,5-dimethylthazolk-2-yl)-2,5-diphenyl tetrazolium bromide (MTT) testing on 5 different cancer cell lines: HCT-116 human colon cancer, MCF7 breast cancer, and the OVCAR-3, IGROV-1, and SKOV-3 human ovary cell lines, and (vi) the contribution of flavonoids and the impact of structure on the structure–activity relationship. In the literature, these flavones are not or are very little studied in terms of their activity against these targets (DPPH, ABTS, AChE, **1**5-lipoxygenase, α-amylase, XOD, SOD, MCF7, OVCAR-3, IGROV-1, and SKOV-3).

## 2. Results and Discussion

### 2.1. Antioxidant Activity

In this part of the analysis, DPPH radical and ABTS radical cation scavenging activity was measured to assess the antioxidant activity of the 20 flavones (Table 1 and Table 2). For the DPPH test, only M7 presented an interesting scavenging activity, at 96.4 ± 0.1% at 100 µM; the IC_50_ value was equal to 5.2 ± 0.2 µM. This result was very interesting compared to vitamin C, which expressed a value of IC_50_ = 2.3 ± 0.2. In a previous study, M7 showed scavenging ability via a DPPH assay at IC_50_ = 21.2 µM [16]. M18 and M8 presented moderate activity (40.8 ± 0.6 and 20.3 ± 4.1% at 100 µM). All the other molecules were inactive at 100 µM. In the ABTS assay, M1, M2, M3, M6, M7, M8, M13, M16, M17, M18, and M19 presented more than 50% inhibition activity (Table 2). M2, M3, M6, M7, M8, and M13 showed the best IC_50_ values at 20.0, 24.0, 24.5, 6.3, 7.3, and 24.5 µM, respectively. M7 presented the most important IC_50_ value, as seen from the DPPH assay. In the literature, M2 has expressed a similarly low antioxidant activity of 3.8 ± 1.0% for the DPPH assay and 14.6 ± 0.1% for the ABTS assay when tested at 147.78 µM [17]. The rest of these molecules have never been tested for antioxidant activity. The antioxidant activity of phenolic compounds depends on their structure, in particular the number and the positions of the hydroxyl groups and the nature of substitutions on the aromatic rings. M7 expressed the best IC_50_ value for the DPPH and ABTS assays, compared to M1, which was not active in the DPPH assay and moderately active in the ABTS assay. M7 presented a supplemented hydroxyl group in the A-ring at position C-6. This specific hydroxyl group position contributes highly to antioxidant activity. The presence of two hydroxyl groups (C-3′ and C-5′) in the B-ring of M8 increased the antioxidant activity in the ABTS assay compared to that of M16. M16 presented methyl groups in C-6 for the A-ring and only a single hydroxyl group in the B-ring (C-3′). M13 and M14 also have two hydroxyl groups, (C-7; C-3′) and (C-5; C-3′), respectively. M13 and M14 were less active compared to M8, in which the hydroxyl group is located at C-3′–C-5′. By looking at the structures of the three most strongly antioxidant compounds (M7, M8, and M18), the only common point between these compounds is the presence of the OH group, which is found in three completely different positions. This highlights the finding that activity is also dependent on the general structure of each molecule (conjugated system, accessibility of OH, disposition in space, etc.).

### 2.2. Biological Activity

#### 2.2.1. Anti-α-Amylase Activity

In total, 14 flavonoids of the 20 that were tested (Table 3) presented interesting anti-α-amylase activity (1.2–37.9 µM). M5 and M13 were the most effective flavonoids with IC_50_ = 1.2 ± 0.1 and 1.4 ± 0.1 µM, respectively better than acarbose IC_50_ = 1.5 ± 0.1 µM. Those molecules presented similar chemical structures, differing just with a hydroxyl group (C-3′) for M13. M7, M9, M10, and M19 showed a good level of activity. M9 and M10 presented analogous IC_50 _= 2.0 ± 0.2 and 2.3 ± 0.1 µM at one-to-one. Those two flavones diverged only in the position of the phenyl group attached to the flavone A-ring (C-5 and C-6, along with C-7 and C-8, corresponding to M9 and M10, respectively). M7 showed a value of IC_50_ = 3.4 ± 0.1 µM; however, M1, with a value of IC_50_ = 36.6 ± 0.2 µM, presented the nearest chemical structure but was 10 times less active. This C-6 hydroxyl group contributes highly to anti-α-amylase activity. M3, M4, and M5, showed the capacity to inhibit α-amylase to the order of 90.3 ± 3.2, 58.4 ± 1.4, and 90.3 ± 3.2%, respectively, at 100 µM. M5 was tested in a previous study by different protocols [18] at 1 mM and demonstrated porcine pancreas α-amylase inhibition at 4.8 ± 3.6%. However, M3 was less active [18] at 4.5 ± 1.1%, whereas M4 was ineffective at 1 mM. M7 demonstrated a value of IC_50_ = 3.4 ± 0.1 µM. In a previous study, M7 displayed 13.2 ± 2.4% of inhibition at 1 mM when α-amylase activity was assayed using the chromogenic substrate, p-nitrophenyl-α-D-maltopentaoside [18]. M13 and M14 having just some differences in hydroxyl-group positions (C-7 and C-5, respectively), were effective against α-amylase (IC_50_ = 1.4 ± 0.1 and 6.7 ± 0.9 µM, respectively), but M13 was four times more active. The C-7 hydroxyl position showed more potential in terms of this activity. Two proposed interactions can be attributed to the inhibitory activity of flavonoids, compared to acarbose activity. The first one involves the hydroxyl groups linked to a flavonoid chemical structure that can form a hydrogen bond with the OH groups present in the enzyme’s active side chains. The second way consists of a conjugated π-arrangement, which can probably form between the A-C ring of the flavonoids and the active site present in α-amylase. Those two interactions can encumber the reaction between α-amylase and starch and, thus, inhibit starch degradation [19].

#### 2.2.2. Effects of Flavonoids on the Inhibition of SOD and XOD

M8, M12, M13, M14, M16, and M17 (Table 3) were very active against XOD (IC_50_ = 0.8–2.3 µM). This flavone series presented similar structures, with two hydroxyl groups differing only in their positions (except in the case of M16 and M17). M12, M13, and M17 showed the best values of IC_50_ = 1.2 ± 0.1, 0.9 ± 0.2, and 0.8 ± 0.1 µM, respectively. Those results were better than the reference, allopurinol (IC_50_ = 1.3 ± 0.1 µM). M8 and M16 displayed similarities in terms of C-3′ hydroxyl position, contributing to enhancing this activity for both flavones. M12 was more effective than M11, differing in hydroxyl number and position and in methoxy group position. The C-4′ hydroxyl group present in M12 and/or C-7 methoxy, which is absent in M11, enhanced this activity. M13 and M14, showing both C-3′ hydroxyl groups, were both active. In M13, the C-7 hydroxyl group helped to improve the IC_50_ value by about four times. M17, with a methoxy group in C-4′ compared to M3, was not active. The methoxy group’s presence in the B-ring diminished the anti-XOD activity. The results for this flavone group were established for the first time in terms of XOD inhibitory activity. The position and number of the hydroxyl group or others function in the flavones to influence anti-XOD activity. This series of molecules was effective in terms of in vitro anti-XOD activity, but this requires supplementary in vivo experiments to confirm their bio-viability, the mechanism involved, their distribution, and if they have health-related side effects or not. In terms of SOD inhibitory activity, only 9 flavonoids were effective; the expressed percentage varied from 52.3 to 80.9% at 100 µM. M7 and M5 revealed the most remarkable values for IC_50 _= 36.5 ± 1.2 and 31.5 ± 0.5 µM, respectively; they presented a common C-7 hydroxyl group. M5 displayed the best results at 74.3 ± 3.1%, whereas M3 and M4 were less effective at 54.8 ± 3.2 and 25.9 ± 2.4%, respectively. The hydroxyl group in the C-7 position contributed markedly to enhancing this activity, compared to the other positions for M3 and M4 in C-6 and C-5. M5, compared to M1, presented a common hydroxyl group in C-7. M1 contained a hydroxyl group in C-5; this group is also present in M15 and negatively affects anti-SOD activity. M17 expressed a higher percentage of inhibition at 80.9 ± 0.2% in 100 µM and IC_50_ > 50 µM. M3 also presented a similar structure (without C-4′), showing 54.8 ± 3.2% of inhibition at 100 µM. Finally, the hydroxyl in the C-7 position is very important for the flavones’ anti-SOD activity.

#### 2.2.3. Anti-AChE Activity 

In total, 10 molecules were active against AChE (50.8–63.9%) at 100 µM (Table 3). M7 was the most potent in terms of this activity, with a value of IC_50_ = 10.2 ± 3.0 µM. M7 presented a supplemented hydroxyl group in position C-6, compared to the less active M1 (45.8 ± 1.3% at 100 µM). The group of M3, M4, and M5 exhibited similar inhibition activities against that enzyme and varied from 40.1 ± 2.8 to 47.7 ± 0.7% at 100 µM. Each hydroxyl, when present on its own, did not allow sufficient activity; however, M7 presented three hydroxyls in C-5-6-7 that contributed to improving anti-AChE activity. Previous in vivo tests on M7 suggested that this flavonoid perfected Aβ1-40-induced dementia in rats and may be a new and promising drug for the treatment of AD [20].

#### 2.2.4. Anti-Inflammatory Activity

Of all the tested molecules, only M7 was effective against the 15-lipoxygenase enzyme (IC_50_ = 38.5 ± 2.7 µM). None of the other flavones exhibited activity at 100 µM. All of the tested flavones (except M7) have never been tested for their anti-inflammatory (15-lipoxygenase) activity in the literature. M7 presented a three-hydroxyl group in C-5-6-7 that contributed to improving anti-AChE activity, compared to the most similar mono or dihydroxyl flavones (M1, M3, M4, and M5), which were ineffective. M7 displayed a specific chemical structure, with two different groups in the A-ring making it highly polar, and B was clearly apolar. M7 is a natural product extracted from *Scutellaria baicalensis* and is also present in herbs used in traditional Chinese medicine against inflammation, hypertension, cardiovascular diseases, and bacterial and viral infection treatments [21]. M7 inhibited 12-lipoxygenase activity (IC_50_ = 1 µM) in a previous report, which may contribute to Alzheimer’s disease prevention [22].

#### 2.2.5. Cytotoxic Activity against Cancer Cell Lines

**Figure 1:**
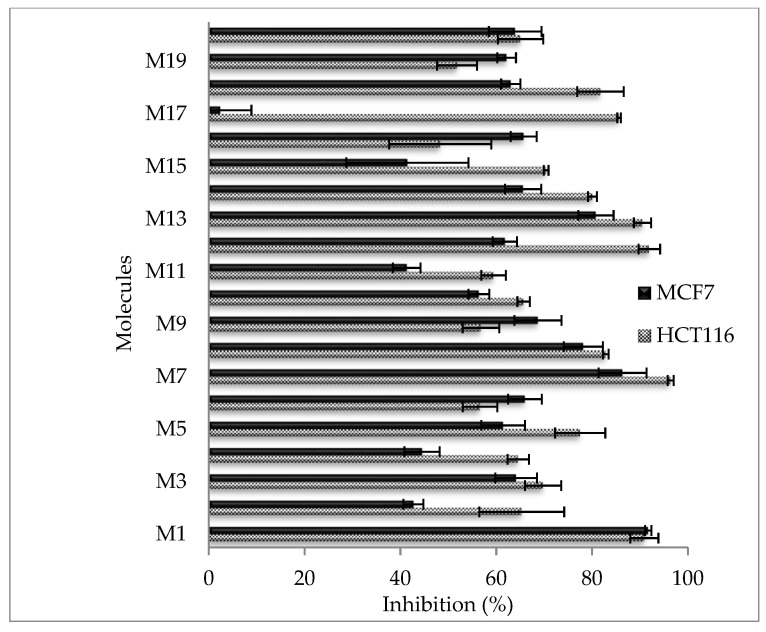
Cytotoxic activity of flavones (100 µM) against the HCT116 and MCF7 cancer cell lines.

**Figure 2:**
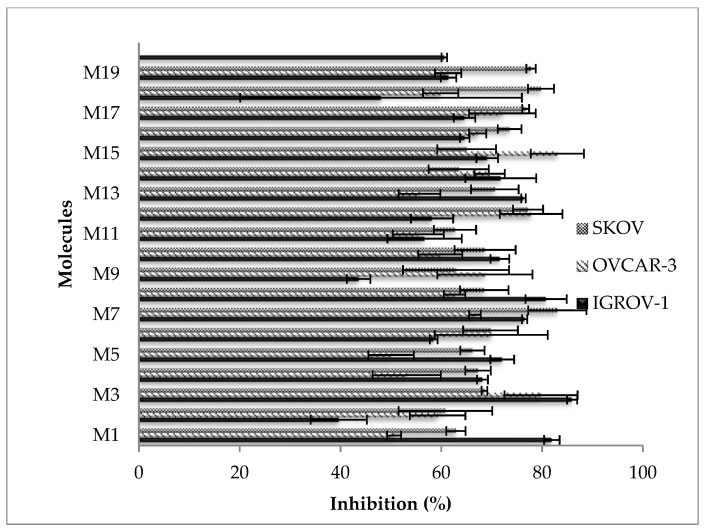
Cytotoxic activity of flavones (100 µM) against the SKOV, OVCAR-3, and IGROV-1 cancer cell lines.

Flavones were tested against various cancer cell lines (Figure 1 and Figure 2). For HCT116 cell lines, the most interesting flavone was M14, with values of IC_50_ = 4.5 ± 0.2 µM (Table 4). M14 (IC_50_ > 50 µM), when compared to M4, exhibited a C-3′ hydroxyl group that contributes to increasing its activity against the HCT116 cancer cell line. Moreover, M14 was nine times more active than M13 (IC_50_ = 39.3 ± 0.8 µM). M14 exhibited a C-5 hydroxyl group, while M13 displayed them in C-7. M7 presented a three-hydroxyl group in C-5-6-7 that contributed to improving cytotoxic activity, compared to M1, M3, M4, and M5, with a value of IC_50_ > 100 µM. M8 (IC_50_ = 33.1 ± 0.1 µM) presented a two-hydroxyl group into C-3′ and C-5′, compared to M16 (with IC_50_ > 100 µM), with a methyl group at C-6 and a hydroxyl group at C-3′. M12 showed a value of IC_50_ = 38.5 ± 1.2 µM compared to M11, where IC_50_ > 50 µM. M12 presented a hydroxyl group in C-4′ and methoxy in C-7, which improved its activity compared to M11, this being the only methoxy group in C-7. M17 displayed an IC_50_ = 40.4 ± 1.2 µM, compared to M3 at IC_50_ > 100 µM. M17 presented a methoxy group at C-4′ that improves activity compared to M3 (IC_50_ > 50 µM). Regarding the MCF7 cell line, 15 flavones expressed interesting activity. M2, M4, M11, and M16 yielded IC_50_ > 100 µM. M1, M7, M8, and M13 displayed the best activity (IC_50_ = 35.9 ± 0.8, 33.1 ± 1.4, 40.4 ± 0.7 and 38.5 ± 1.2 µM, respectively). M1 and M7 presented analogous IC_50,_ with a different supplementary hydroxyl in C6 for M7. M1 contained two hydroxyls (C-5 and C-7) that improved MCF7 cytotoxic activity compared to M3, M4, and M5, which presented only one hydroxyl in C-6, C-5, and C-7, respectively (IC_50_ > 50 µM). The C-6 hydroxyl group, present in both M3 and M7, did not increase this activity. The HCT116 cell line was also compared to M16. Contrary to that in the HCT116 cell line, M13 (IC_50_ = 38.5 ± 1.2 µM) was more active than M14 (IC_50_ > 100 µM). Regarding the OVCAR-3 assay, M3, M12, and M15 were more active (44.7 < IC_50_ < 47.0 µM). The hydroxyl group C-6 in M3 contributed to enhancing this activity (IC_50_ = 44.7 ± 1.2 µM) while M7 (C-5-6-7), M4 (C-4), and M5 (C-7) were less active (IC_50_ > 50 µM). M12 showed IC_50_ = 47.0 ± 1.4 µM compared to M11, with IC_50_ > 50 µM, in the same way as against HCT116. M15 was highly active against the OVCAR-3 cell line and expressed a value of IC_50_ = 45.6 ± 1.4 µM compared to M1 and M4 (their values of IC_50_ > 50 µM). M15 presented a common hydroxyl (C-5) with M1 and M4. This result demonstrates that adding 7-methylbenzyloxy contributes to enhancing this ability to inhibit the OVCAR-3 cell line. In the SKOV-3 test, M7 presented the best value of IC_50_ = 15.6 µM. M7, when compared to M1, M3, M4, and M5 (their values of IC_50_ > 50 µM) presented the same evolution as with the HCT116 cell line. M12 was more active (IC_50_ = 44.7 ± 1.2 µM) compared to M11 (IC_50_ > 100 µM) and was similar to the HCT116 and OVCAR-3 cell lines. M13 was more active (IC_50_ = 42.1 ± 0.7 µM) than M14 (IC_50_ > 100 µM), as with the MCF-7 cell line. M16 was more active than M8, contrary to the HCT116 cell line. M17 showed a value of IC_50_ = 43.8 ± 0.9 µM that was better than for M3 (IC_50_ > 100 µM), as with the HCT116 cell line. The IGROV-1 cell line was used only to determine the percentage of inhibition and IC_50_ was not established, following the loss of this cell line. All flavones were effective against this cell line, except M2, M9, and M18, which were not active (IC_50_ > 100 µM). M1, M3, M5, M7, M8, and M13 were the most active at 100 µM. All these molecules have a common point (except M13) that is related to the presence of both ends of opposite polarity. These results, reported here for the first time, confirm the important contribution of the hydroxyl group flavones (positions and numbers) to cytotoxicity in different cancer cell lines. Their variability of chemical structures begets a selectivity, especially for: (i) M1 (hydroxyl C-5 and C-7), which is selective for the MCF7 cell line; (ii) M3 (hydroxyl C-6) and M15 (hydroxyl C-5 and 7-methylbenzyloxy), which are selective for the OVCAR-3 cell line; (iii) M14 (hydroxy C-5 and hydroxyl C-3′), which are selective for the HCT116 cell line. In the literature, M7 has shown apoptotic activity for the in vitro modulation of cellular metabolic activities [23]. The high levels of activity of M14 against the HCT-116 cell line may contribute to a standard anti-cancer drug. This result needs further in vivo experimentation.

### 2.3. In Silico Docking Study

**Figure 3:**
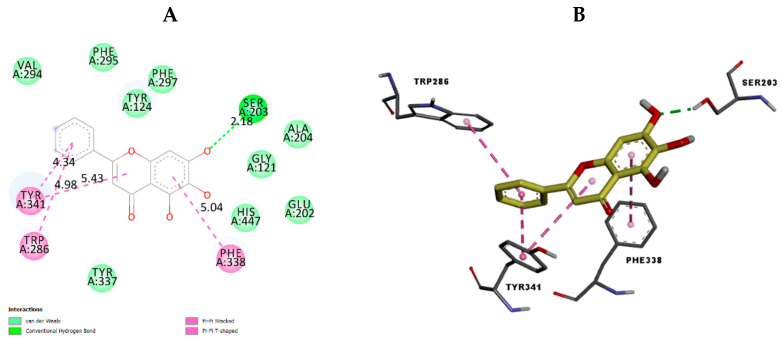
The 2D (**A**) view and 3D (**B**) view of the interaction type of M7 with the surrounding amino acids of acetylcholinesterase.

**Figure 4:**
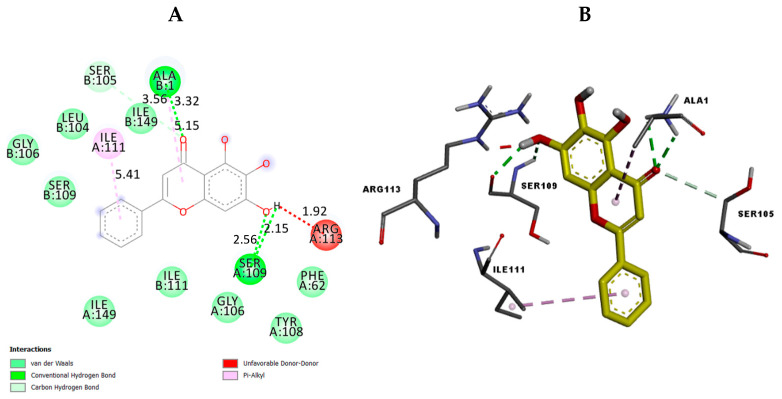
The 2D (**A**) view and 3D (**B**) view of the interaction type of M7 with the surrounding amino acids of superoxide dismutase.

**Figure 5:**
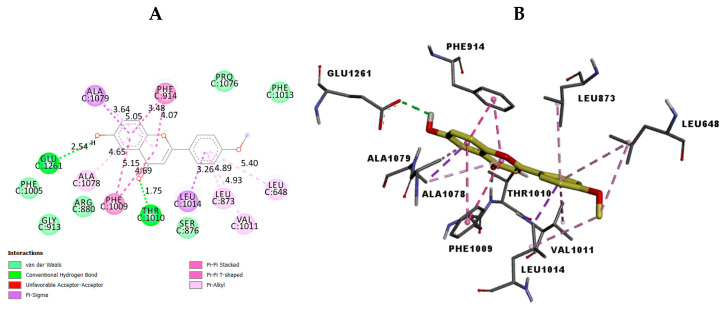
The 2D (**A**) view and 3D (**B**) view of the interaction type of M17 with the surrounding amino acids of xanthine oxidase.

**Figure 6:**
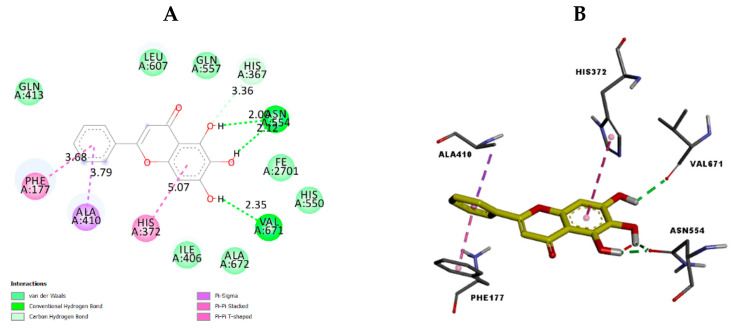
The 2D (**A**) view and 3D (**B**) view of the interaction type of M7 with the surrounding amino acids of 15-lipoxygenase.

**Figure 7:**
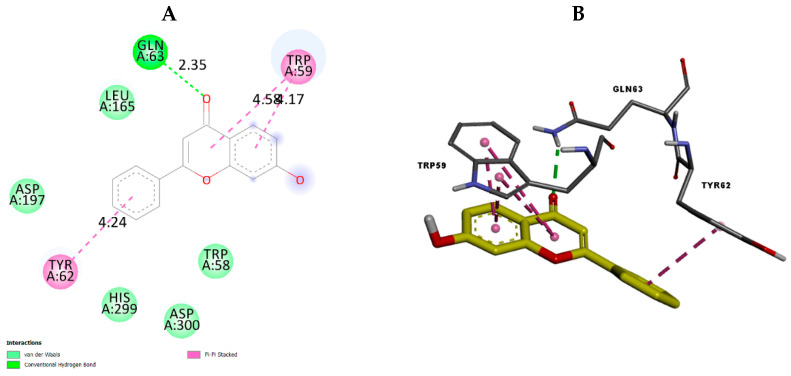
The 2D (**A**) view and 3D (**B**) view of the interaction type of M5 with the surrounding amino acids of α-amylase.

To investigate the possible binding interactions of the flavonoids M1–M20 with the catalytic site of the targets and to elucidate the related inhibitory effect, we performed molecular docking, which is regarded as a powerful computational technique in structure-based drug design. The flavonoids M1–M20 showed different binding energy values against acetylcholinesterase (4EY7), superoxide dismutase (1CB4), xanthine oxidase (1FIQ), 15-lipoxygenase (3V99), and α-amylase (5e0F) (Table 5). We calculated the superimposed RMSD for the docking of the co-crystallized ligand. The results showed good values of 0.951, 0.528, 0.595, and 1.178, respectively, for 4EY7, 5E09, 3V99, and 1FIQ (PDB 1CB4 does not have a co-crystallized ligand). In the current paper, we investigated the interaction modes of the most potent compounds. For acetylcholinesterase, the most potent compound, M7 presented suitable interactions with the different acid residues and the results are shown in Figure 3. The reported results (Table 5) confirmed that compound M7, displaying potent inhibitory effects, fits perfectly at the active site of acetylcholinesterase, with a binding energy of −10.6 kcal mol^−1^. Compound M7 shows one H-bonding between the oxygen (OH group) of the ligand and Ser203 (2.18 Å) (Figure 3). The rings of the ligand show π-π interactions with Tyr341, Trp286, and Phe338. Donepezil, [24] a reference drug, shows interactions with Phe295, Trp86, Trp286, Tyr337, Phe338, and Tyr341. Our results indicated that compound M7 forms interactions with Trp286, Phe338, Tyr341, Phe295, and Tyr337. These interactions are involved in complex stabilization and contribute to its inhibitory effect. For 4EY7, we noted that most compounds have a lower docking score than M7. For M9, M10, M15, M16, and M19, which have a higher docking score, we noted that they have fewer interactions with the key amino acids than M7. Regarding superoxide dismutase, the most potent compound is also M7. Figure 4 presents the numerous interactions of M7 with the protein. The corresponding binding energy is −7.4 kcal mol^−1^. There are two hydrogen bonds formed between the (OH group) of the ligand and Ser105 (2.56 Å and 2.15 Å). There is one hydrogen bond between the oxygen of the carbonyl group and Ala1 (2.15 Å). We also found a π-alkyl interaction with Ile111 and a carbon–hydrogen interaction with Ser105. It was shown that their compounds demonstrate numerous activities with SOD and that they also have interactions with Ser109, Arg113, Tyr108, and Glu 107 [25]. Our compound presented interactions with similar amino acids: Ser109, Arg113, and Tyr108. In the case of 1CB4, M3, M5, M7, M9, and M19, these fitted well into the catalytic site. M7 has more interactions with the catalytic sites than the remaining compounds in this group. The best compound for xanthine oxidase activity was M17. The interactions of M17 with xanthine oxidase are presented in Figure 5. The binding energy is −9.3 kcal mol^−1^. M17 has two hydrogen bonds with Glu1261 (2.54 Å) and Thr1010 (1.75 Å). It also has π-π interactions with Phe914 and Phe1009, along with Pi Alkyl interactions with Leu873, Val1011, Leu648, and Ala1078, as well as Pi sigma interactions with Ala1079 and Leu1014. Joshi et al. [26] found that their two best-performing compounds have interactions with Phe914 and Phe1009 and that a similar type of π-π interaction was observed in the xanthine oxidase active site with allopurinol and salicylic acid [26]. These same interactions are found in our compound with the protein and contribute to its inhibitory effect. In the case of 1FIQ, M8, M12, M13, M14, M16, and M17, which demonstrate good in vitro activity, fit very well with the catalytic site, while M17 presents more interactions than the other compounds. The remaining compounds have different orientations when compared to M17. M7 is the most active compound, with 15-lipoxygenase. It has a binding energy of −7.5 kcal mol^−1^. M7 interactions with the protein are shown in Figure 6. M7 has two hydrogen bonds with Asn554 (2.12 and 2.00 Å) and one hydrogen bond with Val671 (2.35 Å). It has π-π interactions with Phe177 and His372, as well as π-sigma interaction with Ala410. Chaudhry et al. studied the interactions of the compound they developed and found that M7 was the most active LOX inhibitor [27]. They observed that it interacted with the Fe atom and also noticed its interactions with Leu368, Ala672, and Ala410. Our compound showed interactions with similar amino acids: Ala410 and Ala672. It also demonstrates an interaction with the Fe atom. In the case of 3V99, among all the compounds, only M7 fits very well in the active site and has three hydrogen bonds with key amino acids. This condition is not found in the other compounds; only M7 shows biological activity for this protein. M5 is the most active compound for α-amylase. It has a binding energy equal to −8.5 kcal mol^−1^. Figure 7 presents the amino acids of the α-amylase surrounding M5. There is one hydrogen bond between the oxygen of the carbonyl group of the ligand and Gln63 (2.35 Å). There are π-π interactions between the compound and Trp59 and Tyr62. Ramasubbu et al. stated that Glu233, Arg 61, Asp 236, Asp300, Lys 200, Leu165, and Asp197 residues, along with a number of non-polar or aromatic residues, His299, His305, Ile235, Tyr 258, Ala307 Trp58, Trp59, Tyr62, His101, and Ser163, characterized the active site of α-amylase [28]. In another study, Williams et al. noticed that myricetin has an inhibitory effect on α-amylase and was fitted into the catalytic site of α-amylase through four hydrogen-bond interactions with Asp197, His101, and Gln63, as well as through two other hydrophobic interactions with Tyr62 and Leu165 [29]. We notice that there are common amino acids surrounding M5: GLN63, Trp59, Tyr62, Trp58, Asp300, Leu165, Asp197, and His299. For 5E0F, the compounds M5, M7, M9, M10, M13, and M19, which have good in vitro activity, fit very well into the catalytic site, presenting similar hydrogen bonds. M5 demonstrates more interactions with the key amino acids.

## 3. Materials and Methods

### 3.1. Preparation of Molecules

All samples of the molecules (Table 1) were prepared and dissolved in DMSO (2.5%) to a final solution of 100 µM and were aliquoted at the appropriate volumes for each assay, then stored at −20 °C. All the tested molecules were purchased from Sigma Aldrich (Saint-Quentin-Fallavier, France) (chrysin (95082); icariin (56601); 6-hydroxyflavone (411035); 5-hydroxyflavone (H4405); 7-hydroxyflavone (41934); trihydroxyethylrutin (PHR2815); baicalein (465119); 3′,5′-dihydroxyflavone (CDS007027); 3′-hydroxy-b-naphthoflavone (CDS006587); 3′-hydroxy-a-naphthoflavone (CDS006703); 5-hydroxy-3′-methoxyflavone (CDS007060); 4′,5-dihydroxy-7-methoxyflavone; 7,3′-dihydroxyflavone (CDS006791); 5,3′-dihydroxyflavone (CDS006761); 5-hydroxy-7-((3-methylbenzyl)oxy)-2-phenyl-4h-chromen-4-one (R609625); 3′-hydroxy-6-methylflavone (CDS006874); 6-hydroxy-4′-methylflavone (CDS006851); 7-hydroxy-3′,4′,5′-trimethoxy-α-naphthoflavone (S425087); diosmin (D3525); myrcitrin dihydrate (91255)).

### 3.2. Antioxidant Activity and Biological Activities

All tests were performed in triplicate according to the methods specified by El Euch et al. [30].

#### 3.2.1. Spectrophotometry

All optical density assays were performed using a Thermo Scientific Multiskan GO UV/Vis spectrophotometer microplate, allowing wavelength selection for 96-well plates.

##### Free Radical Scavenging Activity

DPPH• (1-1-diphenyl 2-picryl hydrazyl) assay

This method is based on the spectrophotometric assay using the stable radical, DPPH•, as a reagent. First, 20 µL of each sample (4 mg/mL) was mixed with 180 µL of 0.1 mM DPPH• in methanol, in plates, after an incubation of 25 min in the dark. Absorbance was measured at 517 nm. Ascorbic acid was used as the reference compound. The radical-scavenging activities of molecules, expressed as a percentage inhibition of DPPH•, were calculated according to the following formula:

Inhibition (%) = [(A control − A sample)/A control] × 100

where A is the absorbance determined at 517 nm. The absorbance of the solvent and DPPH• radical without the molecule was measured as the control.

ABTS (2,2′-azinobis-3-ethylbenzothiazoline-6-sulfonate) assay

The radical scavenging capacity of antioxidants for the ABTS radical cation was evaluated following this method: ABTS was prepared by adding 7 mM solution of ABTS at pH 7.4 (5 mM NaH_2_PO_4_, 5 mM Na_2_HPO_4_, and 154 mM NaCl) with 2.5 mM potassium persulfate. The mixture was stored at room temperature for 16 h in the dark before use and prepared the evening of the assay. Once diluted with persulfate buffer, the absorbance of the mixture yielded an absorbance value of 0.70 ± 0.05 units at 734 nm. Then, 20 µL of each tested sample was mixed with fresh ABTS solution (180 µL), then the absorbance was measured 6 min after the initial mixing. Ascorbic acid was used as a standard. The capacity of free radical scavenging was expressed as a percentage of the inhibition values. The percentage of inhibition is calculated using the same expression employed in the DPPH assay.

##### Anti-α-Amylase Activity

The α-amylase inhibitory activity (%) value was defined as the percentage decrease in the maltose production rate over the control. Fifty microliters of each sample (at different concentrations of 25, 50, and 100 µg/mL) was tested and added to 50 µL of α-amylase enzyme (1U/mL), then the mixture was incubated for 15 min at 25 °C. Then, we added 100 µL of the substrate starch solution (at 1%, prepared in the same buffer solution under heating at 60 °C and agitation). Finally, we added 100 µL of DNS color reagent solution (96 mM 3,5-dinitrosalicylic acid and 5.31 M sodium potassium tartrate in 2M NaOH) in an Eppendorf tube, putting this in a water bath heated to 100 °C. After 15 min, this mixture was removed from the water bath and diluted with 900 µL of distilled water. The anti-α-amylase activity was determined by measuring the absorbance at 540 nm. Blank incubations were determined as the absorbance produced by samples where the enzyme was replaced with a buffer solution. The total enzyme activity (100%) was assayed with the same procedure, supplementing the sample with DMSO (at 2.5% in the final reaction mixture). Acarbose was used as the positive control. The IC_50_ values were calculated from the mean values of the percentage of α--amylase inhibition data, determined in triplicate.

##### Anti-Xanthine Oxidase Activity

Anti-xanthine oxidase activity was assayed spectrophotometrically under aerobic conditions. First, 50 µL (50 µg/mL) of samples were added to 60 µL of phosphate buffer (pH 7.5) and 30 µL of xanthine oxidase enzyme solution (0.01 units/mL in phosphate buffer; pH 7.5). After pre-incubation at 25 °C for 15 min, the reaction was initiated by the addition of 60 µL of xanthine substrate solution (prepared in the same buffer). The assay mixture was incubated at 25 °C for 5 min. The absorbance was measured at 295 nm. The assay was performed in triplicate. Allopurinol is an effective inhibitor of the enzyme xanthine oxidase (XOD), used here as the positive control. Tests were carried out in triplicate.

##### Anti-Superoxide Dismutase Activity

Pyrogallol auto-oxidation due to oxygen in the air can be inhibited by an enzyme that is naturally present in the body: superoxide dismutase (SOD). Furthermore, variations in the pyrogallol auto-oxidation rate in the presence of SOD were evaluated. Tests were carried out in triplicate. The objective was to find an inhibitor of the auto-oxidation of pyrogallol in the presence of a SOD enzyme solution. Samples were tested at a concentration of 50 µg/mL, followed by incubation for 4 min at 25 °C. The reaction was initiated by the addition of 30 µL of pyrogallol (30 mM) and the absorbance was measured at 325 nm over 4 min. The self-oxidation of pyrogallol was evaluated in the absence of the enzyme. For this, 30 µL of pyrogallol was added to 120 µL of tris buffer (in place of the SOD) (pH 8.5), then followed by the same steps of incubating a positive control (without the molecule); this was performed in the presence of 5% of DMSO to evaluate the percentage of SOD inhibition activity on the auto-oxidation of pyrogallol.

##### Anti-Cholinesterase Activity

The enzymatic activity was assessed via a modified colorimetric Ellman′s method. First, 50 µL of Tris-HCl buffer (pH 8), 25µL of a buffer solution of the sample (50 µg/mL), and 25 µL of an acetylcholinesterase (AChE) enzyme solution containing 2.8 U/mL were mixed. A reaction was then initiated via the addition of 125 µL of 3 mM 5-5′-thiobis-2-nitrobenzoic acid. After incubation for 15 min at 25 °C, 25 µL of a solution of iodide acetylthiocholine15 mM was added to a 96-well microplate. The absorbance of the mixture was measured at 412 nm after 10 min. A control mixture was prepared in a similar way but using DMSO instead of the sample. Inhibition (%) was calculated in the following way:

Inhibition (%) = [(A control − A sample)/A control] × 100,

where (A sample) is the absorbance of the molecule containing the reaction and (A control) is the absorbance of the reaction control. Tests were carried out in triplicate.

##### Anti-Inflammatory Activity

Anti-inflammatory activity was evaluated via the spectrophotometric measurement of the conjugated diene obtained via the oxidation of linoleic acid by 20 µL of the 5-lipoxygenease enzyme (5-Lox). First, 20 µL of the sample was tested individually with sodium phosphate buffer (pH 7.4) containing 5-Lox (500 U) and 60 µL of linoleic acid (3.5 mM), and 170 µL of potassium phosphate buffer solution (0.1 M, pH 7.4). The mixture was then incubated at 25 °C for 10 min and the absorbance was determined at 234 nm. We determined the percentage of inhibition for each sample. Nordihydroguaiaretic acid (NDGA) was used as a positive control. All tests were carried out in triplicate. The percentage inhibition of enzyme activity was calculated using the same method of anti-cholinesterase activity.

##### Cytotoxic Activity

Cell lines

The cancer cell lines used in this study were HCT116 (colorectal carcinoma), IGROV1 (ovarian cancer), OVCAR3 (ovarian cancer), and MCF7 (mammary gland/breast cancer). The first three lines were maintained in RPMI-1640 medium supplemented with 10% FCS, with 2 mM L-glutamine as a complete growth medium, while the base medium for the MCF7 cell line was DMEM (Dulbecco’s Modified Eagle’s medium), with phenol red and 10% fetal bovine serum. Cell lines were maintained in culture and were incubated at 37 °C in an incubator with 5% CO_2_ in a humidified atmosphere. All tests were established at 80% of confluence, and viability was estimated with a trypan blue exclusion assay.

Cytotoxic Activity Evaluation with an MTT Assay

The cytotoxic activities of molecules against cancer cell lines were evaluated using an MTT assay. The MTT colorimetric assay was performed using 96-well plates. Cells were seeded in a 96-well plate, at a concentration of 10 × 10^3^ cells/well for HCT116 and 12 × 10^3^ cells/well for MCF7, IGROV1, and OVAR. Adherent cells were dispatched and incubated at 37 °C overnight in a 5% CO_2_-enriched atmosphere. Cells in the exponential growth phase were incubated at 37 °C for 72 h, with each tested sample at 50 µg/mL. After that, the medium was removed, and cells were treated with 50 µL of 3-(4,5-dimethylthazolk-2-yl)-2,5-diphenyl tetrazolium bromide (MTT) solution (3 mg/mL in PBS) at 37 °C for 20 to 40 min. To dissolve the cells’ mitochondria and thereby precipitate the violet formazan, we added 80 µL of 100% DMSO. Optical density was measured at 540 nm. All tests were established in triplicate. The anti-cancer effect of samples was estimated in terms of growth inhibition percentage. We utilized tamoxifen as an anti-cancer drug solution reference.

#### 3.2.2. Molecular Docking

For the purposes of studying the preferred position of the ligands at the catalytic site of receptors, interactions between the compounds M1-M20 and, respectively, acetylcholinesterase, superoxide dismutase, xanthine oxidase, 15-lipoxygenase, and α-amylase (5e0F) were assessed via molecular docking. The structures were taken from the Protein Data Bank: acetylcholinesterase (PDB code: 4EY7) [31], superoxide dismutase (PDB code: 1CB4) [32], xanthine oxidase (PDB code: 1FIQ) [33], 15-lipoxygenase (PDB code: 3V99) [34], and α-amylase (PDB code: 5e0F) [35]. We took the co-crystallized ligand and all molecules of water from the structures. We assigned polar hydrogens and Gasteiger charges, then AutoDockTools 1.5.2 ADT (AutoDockTools) was used to select a docking grid [36,37]. Each site of the grid box was set at the center of the catalytic site of the corresponding protein; the box sizes were 30 Å * 30 Å * 30 Å for 4EY7, 1FIQ, and 3V99, 42 Å * 62 Å* 42 Å for 1CB4, and 30 * 27 * 24.75 Å for 5e0F. A spacing of 0.375 Å was applied. The compounds, M1–M20, were optimized by a conjugate gradient AMMP [38]. The AutoDock Vina software [39], with an exhaustiveness parameter of 32, was utilized in the calculations. Docking conformation analysis was performed using ADT. The interactions of the receptor with the ligand were examined via the Discovery Studio Visualizer [40].

#### 3.2.3. Statistical Analysis

All data were analyzed using Microsoft Excel for calculating the means, standard deviation, and correlation coefficient (R²).

## 4. Conclusions

The biological activities of the 20 flavones tested herein demonstrated the important role of hydroxyl group positions and numbers in improving their inhibitory activities (antioxidant, anti-SOD, anti-XOD, anti-AChE, anti-inflammatory, and anti-cancer activities against different cancer cell lines). Several molecules display interesting capabilities, depending on the enzyme or cancer cell lines. We can attest that M7 displayed higher capability in the in vitro tests for antioxidant, anti-inflammatory, and anti-Alzheimer’s activity. Studies by means of molecular docking supported the inhibitory potential of the most potent compound for each biological activity. The study of bioactive molecules is a very important point when assessing future in vivo activities.

## Figures and Tables

**Table 1:** The flavone structure list.

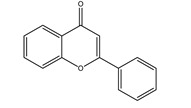 Flavonoids’ Basic Structure			
N°	Name and Structure	N°	Name and Structure
M1	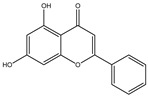 Chrysin (or 5,7-dihydroxyflavone)	M11	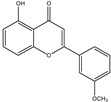 5-hydroxy-3′-methoxyflavone
M2	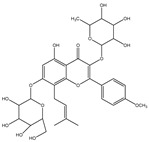 Icariin	M12	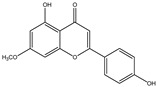 4′,5-dihydroxy-7-methoxyflavone
M3	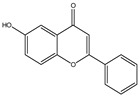 6-hydroxyflavone	M13	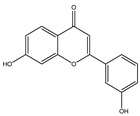 7,3′-dihydroxyflavone
M4	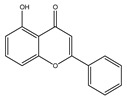 5-hydroxyflavone	M14	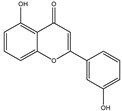 5,3′-dihydroxyflavone
M5	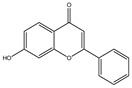 7-hydroxyflavone	M15	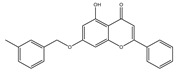 5-hydroxy-7-((3-methylbenzyl)oxy)-2-phenyl-4h-chromen-4-one
M6	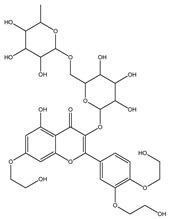 trihydroxyethylrutin	M16	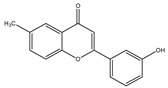 3′-hydroxy-6-methylflavone
M7	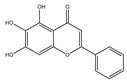 Baicalein (or 5, 6, 7-trihydroxyflavone)	M17	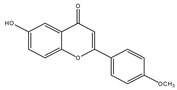 6-hydroxy-4′-methylflavone
M8	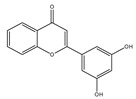 3′,5′-dihydroxyflavone	M18	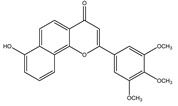 7-hydroxy-3′,4′,5′-trimethoxy-α-naphthoflavone
M9	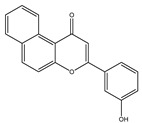 3′-hydroxy-b-naphthoflavone	M19	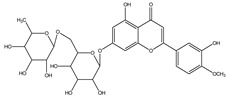 Diosmin (or 3′, 5,7-trihydroxy-4′-methoxyflavone 7-rutinoside)
M10	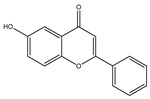 3′-hydroxy-a-naphthoflavone	M20	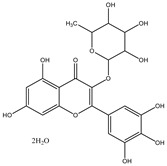 myricitrin dihydrate

**Table 2:** Antioxidant activity (DPPH and ABTS assays) of flavones (percentage at 100 µM and IC_50_ (µM)). na: not active.

	Antioxidant			
Molecule	DPPH Assay		ABTS Assay	
	%	IC_50_	%	IC_50_
M1	na	>100	60.1 ± 3.1	>50
M2	na	>100	87.8 ± 0.9	20.1 ± 0.1
M3	na	>100	95.0 ± 1.2	24.0 ± 0.1
M4	na	>100	16.0 ± 1.5	>100
M5	na	>100	5.9 ± 0.4	>100
M6	na	>100	96.0 ± 0.8	24.5 ± 0.1
M7	96.4 ± 0.1	5.2 ± 0.2	99.4 ± 0.3	6.3 ± 0.1
M8	20.3 ± 4.1	>100	100.1 ± 0.4	7.3 ± 0.1
M9	na	>100	na	>100
M10	na	>100	11.0 ± 1.0	>100
M11	na	>100	17.7 ± 0.7	>100
M12	na	>100	30.3 ± 0.6	>100
M13	na	>100	89.9 ± 0.2	24.5 ± 0.1
M14	na	>100	16.9 ± 0.3	>100
M15	na	>100	25.6 ± 0.7	>100
M16	na	>100	56.7 ± 0.7	>50
M17	na	>100	65.5 ± 0.6	>50
M18	40.8 ± 0.6	>100	50.7 ± 0.7	>50
M19	na	>100	66.6 ± 0.9	>50
M20	na	>100	20.3 ± 3.2	>100
Vitamin C		2.3 ± 0.2		2.2 ± 0.2

**Table 3:** Biological activities of flavones at 100 µM (%) and IC_50_ values. na: not active.

	Anti ACHE		Anti SOD		Anti XOD		Anti 15-Lipoxygenase		Anti α-Amylase	
Molecule	%	IC_50_	%	IC_50_	%	IC_50_	%	IC_50_	%	IC_50_
**M1**	45.8 ± 1.3	>100	41.5 ± 1.8	>100	na	>100	na	>100	59.7 ± 2.0	36.6 ± 0.2
**M2**	19.3 ± 0.3	>100	61.7 ± 1.1	>50	na	>100	na	>100	58.1 ± 2.2	14.1 ± 2.1
**M3**	40.1 ± 2.8	>100	54.8 ± 3.2	>50	na	>100	na	>100	90.3 ± 3.2	10.9 ± 0.1
**M4**	42.3 ± 2.1	>100	25.9 ± 2.5	>100	na	>100	na	>100	58.4 ± 1.4	37.9 ± 0.8
**M5**	47.7 ± 0.7	>100	74.3 ± 3.02	36.5 ± 1.2	na	>100	na	>100	90.3 ± 3.2	1.2 ± 0.1
**M6**	33.7 ± 3.7	>100	53.7 ± 2.1	>50	na	>100	na	>100	49.4 ± 5.1	>100
**M7**	62.0 ± 1.9	10.2 ± 3.0	71.2 ± 2.3	31.5 ± 0.5	na	>100	68.8 ± 2.9	38.5 ± 2.1	96.9 ± 1.7	3.4 ± 0.1
**M8**	49.3 ± 2.2	>100	52.3 ± 0.9	>50	75.3 ± 1.6	2.3 ± 0.1	na	>100	104.8 ± 3.5	14.9 ± 0.3
**M9**	47.9 ± 4.0	>100	19.8 ± 2.9	>100	na	>100	na	>100	86.6 ± 2.3	2.0 ± 0.2
**M10**	54.7 ± 1.9	>50	39.4 ± 1.9	>100	58.8 ± 0.3	>50	na	>100	69.4 ± 1.6	2.3 ± 0.1
**M11**	52.6 ± 4.2	>50	9.4 ± 3.1	>100	na	>100	na	>100	124.8 ± 15.3	8.3 ± 0.2
**M12**	51.0 ± 0.3	>50	42.1 ± 5.3	>100	58.2 ± 3.1	1.2 ± 0.1	na	>100	111.7 ± 0.8	9.0 ± 0.1
**M13**	63.9 ± 3.3	>50	56.6 ± 1.7	>50	64.2 ± 1.2	0.9 ± 0.2	na	>100	91.7 ± 4.9	1.4 ± 0.1
**M14**	55.0 ± 1.9	>50	5.7 ± 2.5	>100	71.3 ± 4.7	2.0 ± 0.1	na	>100	109.6 ± 1.7	6.7 ± 0.9
**M15**	55.3 ± 2.7	>50	26.5 ± 3.3	>100	na	>100	na	>100	46.2 ± 4.4	>100
**M16**	59.1 ± 1.5	>50	27.3 ± 3.2	>100	83.5 ± 3.8	2.0 ± 0.1	na	>100	na	>100
**M17**	50.8 ± 4.1	>50	80.9 ± 0.2	>50	67.7 ± 2.1	0.8 ± 0.1	na	>100	na	>100
**M18**	43.3 ± 1.8	>100	32.7 ± 3.9	>100	19.4 ± 1.0	>100	na	>100	na	>100
**M19**	37.7 ± 4.6	>100	57.8 ± 2.4	>50	45.3 ± 3.2	>100	na	>100	60.6 ± 4.8	3.7 ± 0.3
**M20**	51.8 ± 3.5	>50	42.4 ± 1.1	>100	30.9 ± 2.5	>100	na	>100	na	>100
**Galantamin**	1.4 ± 0.3									
**NDGA**								2.5 ± 0.4		
**Acarbose**										1.5 ± 0.1
**Allopurinol**						1.3 ± 0.1				

**Table 4:** Anti-cancer activity: the IC_50_ (µM) values of flavones tested against HCT 116, MCF 7 SKOV, and OVCAR-3 cancer cell lines (IGROV-1 was not available to establish the IC_50_).

	Cancer Cell Line			
Molecule	HCT116	MCF7	OVCAR-3	SKOV-3
M1	>50	35.9 ± 0.8	>50	>50
M2	>50	>100	>50	>50
M3	>50	>50	44.7 ± 1.2	>50
M4	>50	>100	>50	>50
M5	>50	>50	>50	>50
M6	>50	>50	>50	>50
M7	38.5 ± 1.3	33.1 ± 1.4	>50	15.6 ± 0.1
M8	33.1 ± 0.1	40.4 ± 0.7	>50	>50
M9	>50	>50	>50	>50
M10	>50	>50	>50	>50
M11	>50	>100	>50	>50
M12	38.5 ± 1.2	>50	47.0 ± 1.4	44.7 ± 1.2
M13	39.3 ± 0.8	38.5 ± 1.2	>50	42.1 ± 0.7
M14	4.6 ± 0.2	>50	>50	>50
M15	>50	>50	45.6 ± 1.4	>50
M16	>100	>100	>50	42.1 ± 1.5
M17	40.4 ± 1.2	>50	>50	43.8 ± 0.9
M18	45.0 ± 0.9	>50	>50	37.3 ± 1.0
M19	>50	>50	>50	40.1 ± 0.9
M20	>50	>50	>50	>50
Tamoxifen	1.0 ± 0.2	1.0 ± 0.1	1.4 ± 0.3	1.3 ± 0.1

**Table 5:** Docking binding energies (kcal mol^−1^) of the compounds into the active site of acetylcholinesterase (4EY7), superoxide dismutase (1CB4), xanthine oxidase (1FIQ), 15-lipoxygenase (3V99), and α-amylase (5e0F).

Compound	4EY7	1CB4	1FIQ	3V99	5e0F
M1	−10.4	−7.8	−10.0	−7.6	−8.5
M2	−8.2	−8.6	−7.3	−8.7	−8.6
M3	−10.2	−7.0	−9.6	−7.3	−8.5
M4	−10.3	−7.2	−9.8	−7.7	−8.7
M5	−10.4	−7.9	−9.6	−7.6	−8.5
M6	−9.0	−8.2	−6.5	−8.6	−7.8
M7	−10.6	−7.4	−10.2	−7.5	−8.3
M8	−10.3	−8.1	−10.0	−7.5	−9.1
M9	−12.4	−8.1	−10.2	−8.8	−10.2
M10	−11.2	−8.1	−10.3	−8.7	−10.3
M11	−10.5	−7.5	−9.8	−7.7	−8.7
M12	−10.4	−7.9	−9.5	−7.4	−9.0
M13	−10.5	−8.0	−10.1	−7.7	−8.8
M14	−10.3	−7.7	−9.9	−7.6	−8.9
M15	−11.6	−8.2	−10.9	−9.6	−9.7
M16	−10.9	−7.8	−9.7	−7.9	−9.3
M17	−10.2	−7.2	−9.3	−7.3	−8.3
M18	−10.4	−7.4	−7.6	−8.0	−8.9
M19	−10.9	−8.9	−8.8	−8.8	−9.3
M20	−8.1	−8.8	−6.2	−8.3	−8.3

## Data Availability

The study did not report any data.

## Sample Availability

Samples of the compounds are available from the authors.
